# Erste Daten aus einer digitalen Gesundheits-App für Erektionsstörungen

**DOI:** 10.1007/s00120-022-01872-x

**Published:** 2022-06-20

**Authors:** L. Wiemer, T. Bartelheimer, R. Raschke, K. Miller

**Affiliations:** 1 Pro Uro, Berlin, Deutschland; 2 Charité, Universitätsmedizin Berlin, Berlin, Deutschland; 3 Kranus Health GmbH, München, Deutschland; 4 Urologie am Kurfürstendamm, Berlin, Deutschland; 5 Urologische Facharztpraxis Ralph Raschke, Teltow, Deutschland; 6 Urologische Klinik, Charité – Universitätsmedizin Berlin, Charitépl. 1, 10117 Berlin, Deutschland

**Keywords:** Erektile Dysfunktion, Beckenbodentraining, Kardiovaskuläres Training, Impotenz, Digitalisierung, Erectile dysfunction, Pelvic floor exercise, Cardiovascular training, Impotence, Digitalization

## Abstract

**Ziel:**

Im Rahmen einer systematischen Datenanalyse wurde der Einfluss einer evidenzbasierten digitalen Therapie auf die Erektionsfähigkeit, Patientenaktivierung und Lebensqualität untersucht.

**Methoden:**

Ein 12-Wochen-Programm durchliefen 44 Patienten mit erektiler Dysfunktion (ED) in einer App, bestehend aus Beckenboden-, physiotherapeutischen- und kardiovaskulärem Training. Ergänzt wurde dies durch Anleitungen zur Stressbewältigung, Achtsamkeitsmeditationen und sexualtherapeutischen Inhalten. Zusätzlich erhielten die Patienten Informationen über Ihre Erkrankung und deren Ursachen sowie Tipps zu Ernährung und Risikofaktoren.

Der Altersmedian lag bei 46 (19–75) Jahren. Die Patienten wurden vor Beginn und nach Abschluss mittels IIEF-5-, PAM-13- und QoL-Med-Fragebogen befragt. Bei 27 Patienten konnten die Fragebögen zu beiden Zeitpunkten ausgewertet werden.

**Ergebnisse:**

Nach Absolvierung des Programms ergab sich eine sich eine durchschnittliche Verbesserung von 4,5 Punkten beim IIEF‑5 (*p* < 0,0001). Bei 96 % der Patienten hat sich die Qualität der Erektionen verbessert. 93 % der Patienten zeigten eine Verbesserung der Lebensqualität. Auch bei der Patientenaktivierung zeigte sich eine signifikante Zunahme des durchschnittlichen Gesamtscores.

**Schlussfolgerung:**

Wir konnten zeigen, dass eine multimodale digitale Anwendung zum Selbstmanagement signifikante Verbesserungen der erektionsbezogenen Lebensqualität, der Patientenaktivierung und des Erektionscores bewirkt. Wir sehen, dass Ergebnisse analoger Studien in einer digitalen Gesundheitsanwendung reproduziert werden konnten. Digitale Lösungen erleichtern die Umsetzung der Leitlinienempfehlungen und helfen, Patienten besser in Ihre Behandlung einzubeziehen.

## Abstract (en)

**Purpose:**

In a systematic data analysis, we evaluated the influence of a digital health app on erection scores as well as life quality and patient activation in a group of patients with erectile dysfunction.

**Methods:**

In all, 44 participants took part in an evidence-based program for patients with erectile dysfunction. The in app 12-week program included pelvic floor exercises and physiotherapeutic and cardiovascular training. In addition, there where sessions on mindfulness and sexual therapy as well as useful information about erectile dysfunction and its causes, nutrition, and risk factors. The median age was 46 years (19–75 years). All patients answered IIEF‑5, PAM-13 and QoL-Med questionnaires at the beginning and the end of the program. A total of 27 questionnaires could be evaluated at both times.

**Results:**

The average improvement in IIEF‑5 score was 4.5 points (*p* < 0.0001). 96% of patients showed overall improvement of erection scores. Improvement in life quality was shown in 93% of participants. Moreover, there was a significant increase in patient activation scores.

**Conclusion:**

We were able to show that a multimodal digital app for self-management of erectile dysfunction improved not only erection scores but also life quality and patient activation. We concluded that it is possible to reproduce results of analog studies in a digital setting. Digital solutions can help to include patients in their treatment and to put guideline suggestions into practice.

Erektionsstörungen sind ein häufiges Problem. Obwohl eine leitliniengerechte Therapie eine Risikofaktorminimierung und eine Veränderung des Lebensstils beinhaltet, kommt vielen Patienten primär nur eine medikamentöse Therapie zu [1]. Hinzu kommt, dass Erektionsprobleme ein großes Tabu sind und nur ein kleiner Teil der Betroffenen überhaupt zum Arzt geht. Eine digitale Gesundheitsanwendung soll nun helfen, die Empfehlungen der Leitlinien besser umzusetzen.

In Deutschland alleine sind geschätzt 6–8 Mio. Männer von Erektionsstörungen betroffen. Die genaue Anzahl ist dabei unklar, da Erektionsstörungen nach wie vor ein Tabuthema sind und viele Männer nicht über ihre Probleme sprechen [2]. So konsultieren nur etwas mehr als die Hälfte der betroffenen Männer einen Arzt oder eine Ärztin [3]. Was wir wissen: Die Prävalenz von Männern, die an Erektionsstörungen leiden, nimmt mit dem Alter zu. Fast jeder zweite Mann über 50 Jahre ist betroffen [4], aber immerhin auch schon 8 % der 20- bis 29-jährigen Männer [3] haben mit Erektionsproblemen zu tun. Oftmals entsteht eine erektile Dysfunktion (ED) multifaktoriell. Das Zusammenspiel zwischen Gefäßen, Nerven, Hormonen und Psyche ist Voraussetzung für eine intakte Erektion. Jede Störung in diesem System kann zu Erektionsstörungen führen.

Mögliche Ursachen einer Erektionsstörung sind [5]:

- vaskulär (arteriell [z. B. Arteriosklerose] oder venös [z. B. venöse Leckage]),
- neurogen (zentral (z. B. MS, Parkinson) oder peripher [z. B. Diabetes mellitus, Polyneuropathie, Zustand nach radikaler Prostatektomie]),
- hormonell (z. B. Hypogonadismus, endokrine Störungen),
- anatomisch (z. B. Induratio penis plastica),
- psychisch (z. B. Stress, Versagensängste, partnerschaftliche Probleme),
- Nebenwirkungen, die durch die Einnahme von Medikamenten und anderen Substanzen entstehen können.

## Erektionsstörungen als Vorbote von kardiovaskulären Ereignissen

Wesentlich ist eine hohe Koinzidenz von allgemeinmedizinischen Krankheitsbildern (v. a. Diabetes mellitus, Fettstoffwechselstörungen, Herzerkrankungen und Bluthochdruck; [6]). Da die tiefen Penisarterien (Aa. penis profundae) nur einen Innendurchmesser von 1–2 mm haben, kommt es hier zu einer frühzeitigen Manifestation von arteriosklerotischen Veränderungen. Eine Erektionsstörung kann folglich als frühes Warnsystem für Erkrankungen wie koronare Herzerkrankung (KHK), Myokardinfarkt, arterieller Hypertonie, Apoplex oder einer peripheren Verschlusskrankheit (pAVK) gesehen werden [7, 8]. Eine erektile Dysfunktion tritt in der Regel etwa 5 bis 7 Jahre vor einem Herzinfarkt oder Schlaganfall auf [9].

Isidori et al. konnten zeigen, dass eine erektile Dysfunktion eng korreliert mit einem erhöhten kardiovaskulären Risiko und abhängig ist von dem kombinierten Effekt von Hyperglykämie, Bluthochdruck, arteriellen Veränderungen und Hypogonadismus [10].

Kardiovaskuläre Erkrankungen und Erektionsstörungen haben Risikofaktoren wie Adipositas, Diabetes mellitus, eine Dyslipidämie, Bewegungsarmut und Rauchen gemeinsam. Von den ungesunden Lebensstilfaktoren spielt die physikalische Inaktivität die größte Rolle [11].

## Psyche und Erektion

Psychologische Faktoren spielen bei Erektionsstörungen eine große Rolle. Sie nehmen Einfluss auf die Genese, auf Lebensqualität und Partnerschaft, die diagnostische Evaluation und die Effektivität der Therapie. Beinahe 90 % der Männer mit schweren Depressionen haben auch Erektionsprobleme. Umgekehrt haben Männer mit Erektionsproblemen auch ein ca. 3fach erhöhtes Risiko, eine Depression zu erleiden [12].

Stress und Versagensangst können zu einem Teufelskreis werden. Das Erleben von Potenzstörungen kann zu Verhaltensänderungen führen, die zu Angstzuständen und zum Auftreten von Erektionsstörungen beitragen. Bei der Verursachung psychogener Erektionsstörungen handelt es sich um ein komplexes Geschehen, in das innerpsychische, partnerbezogene und lebensgeschichtliche Faktoren involviert sind.

Eine ED kann folglich die physische und psychische Gesundheit stark beeinflussen und hat einen starken Einfluss auf die Lebensqualität des Betroffenen und der Partnerin oder des Partners.

## Leitlinienempfehlungen sind nur schwer umzusetzen

Sowohl die deutsche neurologische Leitlinie als auch die europäische urologische Leitlinie nennen die ursächliche Therapie mit einer Lebensstiländerung und Reduktion von Risikofaktoren (u. a. mehr Bewegung, Körpergewichtsreduktion, Nikotinverzicht) sowie eine Behandlung psychischer oder anderer kausaler Ursachen als wichtigste und primäre Therapie [5, 13].

Leider fehlt es im medizinischen Alltag oft an der Zeit und an der Vergütung diese Empfehlungen umzusetzen. Selbst die Informationsübermittlung über zugrunde liegende Ursachen und assoziierte Erkrankungen oder verschiedene Therapiemöglichkeiten kommt daher oft zu kurz. Die Verschreibung eines Phosphodiesterase-5(PDE-5)-Inhibitors ist mit Abstand die häufigste Ersttherapie, obwohl ein ursächlicher Effekt nicht nachgewiesen werden konnte [1, 14].

## App als neuartige Therapieerweiterung

Eine digitale Gesundheits-App soll die Umsetzung der Empfehlungen der Leitlinien erleichtern und dem Patienten eine Einbindung in die Behandlung seiner Erkrankung ermöglichen. Die App ist für alle Patienten mit Erektionsstörungen zugelassen, niederschwellige körperliche Tätigkeiten sollten durchführbar sein.

Mit dem Inkrafttreten des Digitale-Versorgung-Gesetzes (DVG) ist es möglich, digitale Gesundheitsanwendungen (DiGA) auf Kassenrezept zu verschreiben. Bislang gibt es 31 Apps (Stand 19.03.2022), welche auf diese Weise von der GKV (Gesetzliche Krankenversicherung) verschrieben werden können. Mit Kranus Edera steht seit 17.12.2021 die erste urologische DiGA zur Verfügung (https://diga.bfarm.de/de/verzeichnis?category=%5B%2286%22%5D).

## Fragestellung

Ziel dieser systematischen Datenanalyse war es, den Einfluss einer digitalen Gesundheitsanwendung (Kranus Edera) auf die Erektionsfähigkeit, die Patientenaktivierung und die Lebensqualität der Patienten zu evaluieren.

## Methodik

## Ablauf und Inhalte der digitalen Therapie

Zu Beginn erhielten alle Patienten einen medizinischen Anamnesebogen und Fragebögen zu Erektionsfähigkeit und PROM („patient reported outcome measures“). Eingeschlossen wurden alle Männer über 18 Jahre mit einer erektilen Dysfunktion (IIEF-5 < 21). Exklusionskriterien waren die Unfähigkeit physisch am Programm teilzunehmen oder das Vorliegen eines mittleren bis hohen kardiovaskulären Risikos nach der Princeton-Klassifikation [15]. Die Princeton-Klassifikation evaluiert das Risiko von Patienten mit ED akute kardiovaskuläre Ereignisse bei körperlicher Aktivität zu erleiden. Weiteres Ausschlusskriterium war eine neu begonnene Therapie mit PDE-5-Inhibitoren oder Prostaglandinen.

Nach Einschluss begann das 12-Wochen-Selbstmanagementprogramm in der App.

Der Teilnehmer erhielt dort Informationen und Hintergrundwissen zu seiner Problematik und absolvierte ein Programm aus Beckenbodentraining, kardiovaskulärem Training und mentalen und sexualtherapeutischen Übungen. Bei Letzteren handelt es sich zum einen um Achtsamkeitsübungen zur Stressreduktion, zum anderen um Übungen zur Körperwahrnehmung nach dem Hamburger Modell, auch als Sensate Focus bekannt. Das Beckenbodentraining erfolgt mittels eines „digitalen Trainers“, der sowohl haptische, als auch visuelle Signale gibt, dabei werden Schnell- und Haltekraftübungen trainiert. Ergänzt wird dies durch physiotherapeutische Übungen, die einem in Videosequenzen vorgegeben werden.

Zur Einstufung des Fitnesslevels und Erfassung möglicher Einschränkungen (Knieprobleme etc.) erfolgt vor Programmstart eine Anamnese. Anhand derer und eines Algorithmus wird dem Patienten eine Sportart (Laufen, Walken, Radfahren) zum kardiovaskulären Intervalltraining empfohlen. Der Trainingsplan nimmt innerhalb der 12 Wochen an Komplexität zu, passt sich je nach Patientenfeedback aber an (Tab. 1). Nach Abschluss des Programms wurden die Teilnehmer erneut mittels Fragebögen befragt.

**Table 1:** 

Kardiovaskuläres Intervalltraining (mögliche Sportarten: Laufen, Walken, Radfahren)
Physiotherapeutische Übungen
Beckenbodentrainer (Schnell- und Haltekraft)
Mentale Übungen
Sexualtherapeutische Übungen
Wissensvermittlung u. a. über: – Erektionsstörungen, ihre Ursachen und Behandlung – Gesunde Lebensstilveränderungen und Ernährung – Psychische Vorgänge – Partnerschaft – Präventionsmaßnahmen urologischer Erkrankungen

### Fragebögen

Neben der medizinischen Anamnese wurden folgende Fragebögen zur Evaluation der Erektionsfähigkeit und der PROM verwendet:

#### IIEF-5

Der International Index of Erectile Function (IIEF-)Score ist ein validierter Fragebogen, der zur Diagnose einer erektilen Dysfunktion und deren Schweregrad entwickelt wurde. Der in seiner Langform aus 15 Fragen bestehende Fragebogen wurde durch ein internationales Panel für die klinische Studien entwickelt und enthält neben Fragen zur sexuellen Funktion, Fragen zu sexuellem Verlangen sowie sexueller und allgemeiner Zufriedenheit. Die gekürzte Version legt den Fokus auf die erektile Funktion und eignet sich daher gut für die Diagnose einer erektilen Dysfunktion und die Einteilung in Schweregrade sowie den Prä-post-Vergleich von Interventionen. (Tab. 2; [16, 17]).

**Table 2:** 

22–25 Punkte: keine erektile Dysfunktion
17–21 Punkte: milde erektile Dysfunktion
12–16 Punkte: milde bis moderate erektile Dysfunktion
8–11 Punkte: moderate erektile Dysfunktion
< 8 Punkte: schwere erektile Dysfunktion

*IIEF* International Index of Erectile Function

#### Qol-Med-Fragebogen

Veränderungen der Lebensqualität sind bei ED von großer Bedeutung. Der, aus 18 Fragen bestehende, psychometrische QoL-Med-Fragebogen erhebt die krankheitsbezogenen Auswirkungen der ED auf Lebens- und Partnerschaftsqualität [19, 20]. Die Werte der Einzelfragen (1–4) werden aufsummiert und auf eine Skala von 0 (niedrigste Lebensqualität) bis 100 (beste Lebensqualität) transformiert.

#### Patient-activation-measure-(PAM-)13-Fragebogen

Der PAM-13 ist ein Fragebogen, um die sog. Patientenaktivierung zu erfassen, das Ausmaß, in welchem sich ein Patient aktiv an der Gestaltung seiner Behandlung beteiligt und wie souverän er mit ihr umgehen kann [21, 22]. Jede Frage wird auf einer Likert-Skala von 1–4 präsentiert. Die Gesamtsumme der Fragenscores wird auf eine Skala von 0 (niedrigste Aktivierung) bis 100 (stärkst mögliche Aktivierung) transformiert.

## Design und Statistik

Patienten konnten bis Mai 2021 eingeschlossen werden und absolvierten das Programm bis Ende August 2021. Es handelt sich um eine PMCF(„post market clinical follow-up“)-Analyse eines zertifiziertes Medizinproduktes nach MDD (Richtlinie 93-42-EWG).

Die Datenanalyse erfolgte mit der Software SAS v9.4 (SAS, Cary, NC, USA). Die statistische Auswertung wurde mittels verschiedener deskriptiver Methoden durchgeführt. Als parametrisches Verfahren zum Vergleich der zwei Zeitpunkte (Woche 0/Woche 12) wurde der gepaarte t Test („paired t test“) bei abhängigen Daten verwendet. Ein *p*-Wert < 0,05 wurde als statistisch signifikant gewertet.

## Ergebnisse

### Patientenkohorte

Die Patientenkohorte bestand aus 44 Probanden im Alter von 19–75 (Durchschnittsalter: 46 ± 16,5) Jahren, die 12 Wochen an dem digitalen Programm in der Gesundheits-App teilgenommen hatten. Kardiovaskuläre Risikofaktoren wie eine Hypertonie und ein Diabetes mellitus zeigten sich in einem der Prävalenz in Deutschland repräsentativen Umfang. Eine Hypercholesterinämie bestand bei 22 % der Patienten (Tab. 3).

**Table 3:** 

Alter	46 (19–75)
BMI	25,4 (19,4–33,08)
Hypertonie	33 %
Hypercholesterinämie	22 %
Diabetes mellitus	7 %
Nikotinabusus	15 %

Prozentzahlen zeigen Anteil der befragten Teilnehmer

*BMI* Body Mass Index

Neun Teilnehmer füllten die Fragebögen am Zeitpunkt 2 (nach 12 Wochen) nicht aus, 8 Teilnehmer wurden aus der Analyse ausgeschlossen. Gründe dafür waren bei 4 Patienten keine Programmaktivität aus persönlichen Gründen (Tod der Ehegattin, länger anhaltende Krankheit, Zeitmangel), 2 Patienten hatten bei Einschluss einen IIEF > 21, 2 hatten technische Probleme und konnten daher nicht die App benutzen. So konnten letztendlich Fragebögen von 27 Patienten ausgewertet werden.

**Figure 1:**
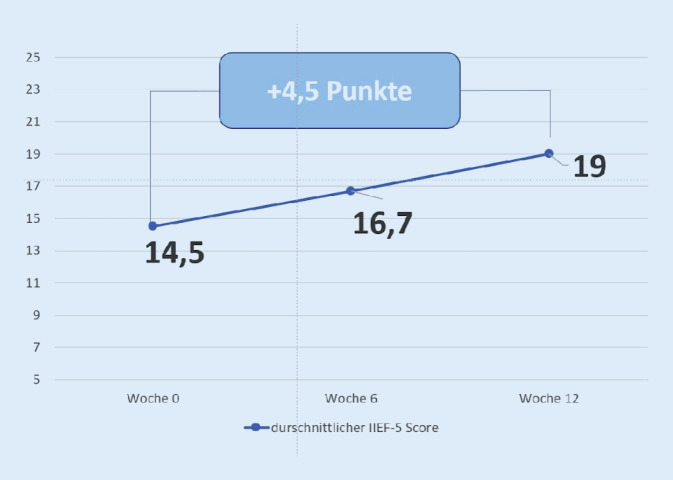

Nach Absolvierung des digitalen Programms in der App berichteten 96 % der Patienten von einer Verbesserung des IIEF-5-Scores. Der durchschnittliche Score verbesserte sich von 14,48 auf 19 (±2,81; *p* < 0.0001, d = 1,61; Abb. 1). Keiner der eingeschlossenen Patienten hatte vor Programmbeginn einen IIEF > 21. 30 % der Patienten erreichten nach den 12 Wochen einen IIEF über 21, womit nach Definition keine erektile Dysfunktion mehr vorliegt.

**Figure 2:**
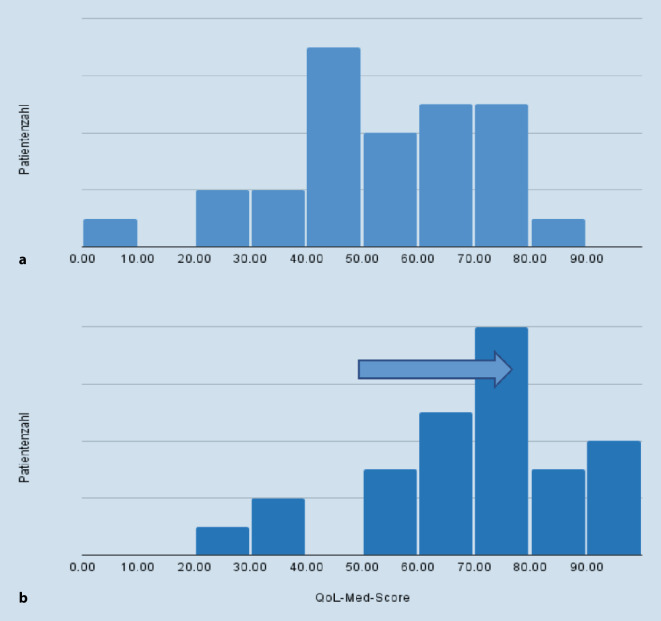

Die überwiegende Mehrheit der Teilnehmer gab eine Verbesserung der Lebensqualität am Programmende an (93 %). Es ergab sich eine relative Verbesserung um 32,4 % beim Prä-post-Vergleich des QoL-Med-Fragebogen. Die Rechtsverschiebung der Scorewerte der Teilnehmer nach Durchführung des Programms spiegelt die Verbesserung der Gesamtheit der Kohorte wider (Abb. 2). Der durchschnittliche Scorewert (auf einer Skala von 0–100) verbesserte sich von 53,8 auf 71,2 (±20,83; *p* = 0.0002, d = 0,78).

**Figure 3:**
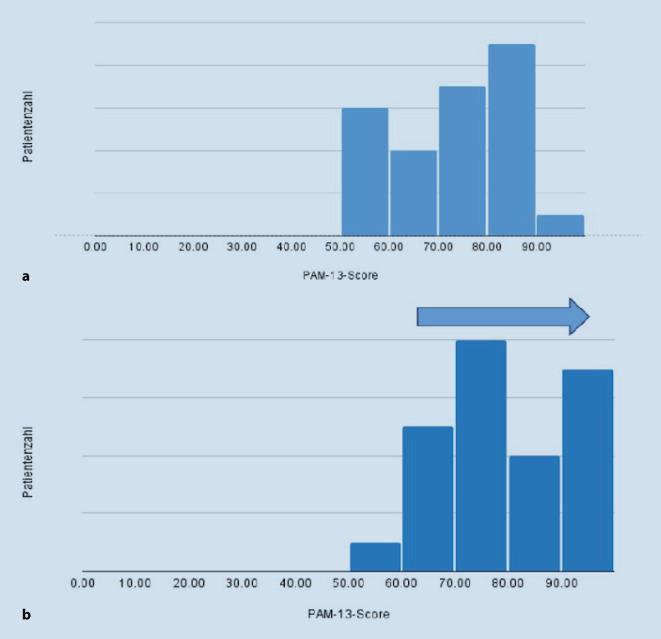

Auch bei der Befragung zur Patientenaktivität zeigte sich bei der Mehrheit der Patienten eine Verbesserung im Ergebnis (70 %). Der mediane PAM-13-Gesamtscore nach Programmabschluss lag bei 81,4. Der PAM-13-Gesamtscore wird auf verschiedene Aktivierungslevel (1–4) übertragen. Ein Wert von 81,4 entspricht Aktivierungslevel 4 und zeigt an, dass die Patienten verstanden haben, dass sie selbst einen aktiven Part in ihrer Behandlung übernehmen müssen und sie sich befähigt fühlen die Behandlung in schwierigen oder stressigen Situationen fortzuführen. Auch bei der Betrachtung der Verteilung fiel eine Rechtsverschiebung (also eine Gesamtverbesserung der Ergebnisse) auf (Abb. 3). Die Veränderung des durchschnittlichen Wert vom Start von 73,22 auf 81,4 am Ende war statistisch signifikant (*p* = 0.0006, d = 0,75; Tab. 4).

**Table 4:** 

	Mittelwert	h	Unteres 95 %-KI	Oberes 95 %-KI	Min	Median	Max
*IIEF‑5 baseline*	14,48	4,23	12,81	16,15	5,00	14,00	21,00
*IIEF‑5 Woche 12*	19,00	4,18	17,35	20,65	9,00	19,00	25,00
*QoL-MED baseline*	53,77	19,10	46,22	61,33	5,56	53,70	88,89
*QoL-MED Woche 12*	71,19	19,46	63,50	78,89	22,22	74,07	100,00
*PAM-13 baseline*	73,22	13,14	68,02	78,42	51,28	74,36	92,31
*PAM-13 Woche 12*	81,39	12,21	76,56	86,22	58,97	79,49	100,00

*IIEF* International Index of Erectile Function, *KI* Konfindenzintervall, *Min* Mindestwert = kleinster Wert, *Max* maximaler Wert = höchster Wert, *PAM* „patient activation measure“, *QoL-Med* „quality of life measure“

## Diskussion

Erektionsstörungen sind aufgrund der hohen Anzahl betroffener Männer und der weitreichenden negativen Auswirkungen dieser Störungen ein bedeutsames Gesundheitsproblem, welches multifaktorielle Ursachen hat und einen multimodalen Behandlungsansatz benötigt. Eine ursächliche Therapie und Risikofaktormodifizierung werden sowohl von der European Association of Urology (EAU) als auch den deutschen (neurologischen) Leitlinien empfohlen [5, 13].

Studien konnten belegen, dass körperliche Aktivität und Beckenbodentraining positive Auswirkungen auf die erektile Funktion haben. Auch die sexualtherapeutischen Ansätze und Entspannungs-sowie Achtsamkeitsübungen sind wissenschaftlich gut belegt. In einer Metaanalyse von 7 randomisierten Studien mit insgesamt 478 Teilnehmern konnte gezeigt werden, dass eine signifikante Assoziation zwischen körperlicher Aktivität und einer Verbesserung der Erektion besteht. Die Verbesserung im Erektionsscore (sechs Studien benutzten den IIEF-5-, eine den IIEF-6-Fragebogen) wurde mit 3,85 (2,33–5,37 Punkten; 95 %-Konfidenzintervall [KI]) angegeben [23]. Aerobes Training hat dabei noch weitere Vorteile, die gerade Patienten mit kardiovaskulären Risikofaktoren zu Gute kommen: verbessertes Herzzeitvolumen, verbesserte körperliche Belastung sowie Verbesserung des glykämischen Profils und Hyperlipoproteinämie. Dies führt zu vermindertem oxidativen Stress und erhöht penile Stickoxid (NO)-Level [24].

Ein weiterer ursächlicher Ansatz ist Beckenbodentraining. Es wird davon ausgegangen, dass die Kontraktion der oberflächlichen Beckenbodenmuskulatur zu einem Anstieg des intracavernösen Druck führt und so den venösen Abfluss über die Crura penis verlangsamt. Vier systematische Reviews beschäftigten sich mit der Frage der Wirksamkeit von Beckenbodentraining im Zusammenhang mit einer erektilen Dysfunktion. Alle kommen zu dem Schluss, dass Beckenbodentraining wirksam als Therapie der ED ist [25, 26].

Die psychosozialen Auswirkungen von Erektionsstörungen sind nicht zu unterschätzen, eine Psycho- oder Sexualtherapie kann und sollte eine sinnvolle Ergänzung der Therapie der erektilen Dysfunktion darstellen. Nur ist leider der Zugang zu geeigneten TherapeutInnen oft begrenzt. Studien konnten zeigen, dass Techniken wie Achtsamkeitstraining, Körperwahrnehmung, Stressbewältigungsansätze und Mediation nicht nur als Teil der Sexualtherapie, sondern auch losgelöst davon, gute Erfolge bei Erektionsstörungen erzielen konnten [27, 28].

Wir konnten eine durchschnittliche Verbesserung von 4,5 Punkten auf der IIEF-5-Skala nach Durchlaufen des 12-Wochen-Programms feststellen. Dieser signifikante Unterschied könnte durch die Kombination der genannten Ansätze, zusammen mit edukativen Inhalten, entstanden sein. Allerdings ist das „reporting“ der bestehenden Studien uneinheitlich, nicht alle nehmen den IIEF als Standard. Auch die Interventionszeiträume unterscheiden sich z. T. stark und die Trainingseinheiten oder -module sind oft uneinheitlich. Ein additiver Effekt der einzelnen Therapiebausteine kann so nur begrenzt bemessen werden.

Hauptlimitation dieser Datenanalyse ist die begrenzte Teilnehmerzahl. Weiterhin konnten von 9 Teilnehmern keine Follow-up-Daten erhoben werden. Sicherlich besteht auch ein Bias in der Patientenkohorte. Man könnte davon ausgehen, dass Patienten die an einem „Selbstmanagement“-Programm teilnehmen an sich zugänglicher für Gesundheitstherapien sind und frühzeitiger medizinische Hilfe aufsuchen. Eine hohe Bereitschaft einen aktiven Part in der Behandlung einzunehmen zeigt sich auch am guten durchschnittlichen Startwert beim PAM-13-Fragebogen. Aber auch diese Patienten zeigen weiteres Verbesserungspotenzial, was sich in der allgemeinen Rechtsverschiebung der Gesamtscores und der signifikanten durchschnittlichen Verbesserung unserer Analyse zeigt. Weiterhin wird bei der untersuchten Kohorte wahrscheinlich eine gewisse digitale Affinität vorhanden sein. So könnte man spekulieren, dass besonders junge Patienten an einer digitalen Therapie interessiert sind. Unsere Daten zeigten allerdings, dass über 40 % der Patienten über 50 Jahre alt waren, nur 20 % der Teilnehmer waren unter 30 Jahre alt. Gegebenenfalls ist aber auch ein auch besonders hoher Leidensdruck vorhanden, was die Patienten zugänglicher für neue Therapiemöglichkeiten macht. 26 % der Patienten unserer Kohorte hatten vor Programmbeginn eine schwere oder moderate erektile Dysfunktion. Erfreulicherweise fielen nach Programmabschluss nur noch 4 % in diese Kategorie, wobei kein Teilnehmer mehr eine schwere ED angab.

Ein digitales Programm kann nicht alle ursächlichen Faktoren von Erektionsstörungen beheben. So kann es zwar in gewissem Maße auch bei Hormonstörungen unterstützen, wird aber beispielsweise eine Testosteron-Therapie bei Hypogonadismus nicht ersetzen können. Auch Patienten mit ausgeprägten psychischen Beschwerden werden nicht nur mit einer digitalen Therapie auskommen. Um geeignete Patienten zu selektieren benötigt es eine gute Zusammenarbeit mit der behandelnden Ärzteschaft als auch mit Therapeutinnen und Therapeuten.

## Schlussfolgerung

Unsere Daten zeigen eine signifikante Zunahme der Erektionsfähigkeit durch Teilnahme an einem digitalen Selbstmanagementprogramm. Es konnte gezeigt werden, dass die Patienten zusätzlich deutlich an Lebensqualität gewinnen und auch eine wesentliche Verbesserung in der Patientenaktivierung entsteht. Wir sehen, dass Ergebnisse analoger Studien zu Beckenbodentraining und körperlicher Aktivität in einer digitalen Gesundheitsanwendung reproduziert werden konnten. Die Leitlinien empfehlen eine ursächliche Therapie mit Modifizierung von Risikofaktoren. Ganzheitliche digitale Lösungen erleichtern die Umsetzung der Leitlinienempfehlungen und helfen Patienten besser und langfristiger in Ihre Behandlung miteinzubeziehen. Die digitale Unterstützung sollte als Teil der Standardtherapie der ED für jeden Patienten in Betracht gezogen werden.
